# SYNGAP1 Syndrome and the Brain Gene Registry

**DOI:** 10.3390/genes16040405

**Published:** 2025-03-30

**Authors:** Melissa R. Greco, Maya Chatterjee, Alexa M. Taylor, Andrea L. Gropman

**Affiliations:** 1 Division of Neurogenetics and Neurodevelopmental Pediatrics, Children’s National Hospital, Washington, DC 20010, USA; melissag22@vt.edu (M.R.G.); mchatterje@childrensnational.org (M.C.); ataylor4@childrensnational.org (A.M.T.); 2 St. Jude Children’s Research Hospital, Memphis, TN 38105, USA

**Keywords:** SYNGAP1, brain gene registry, neurodevelopmental disorder, intellectual disability

## Abstract

Background: The human brain relies on complex synaptic communication regulated by key genes such as *SYNGAP1*. *SYNGAP1* encodes the GTPase-Activating Protein (SYNGAP), a critical synaptic plasticity and neuronal excitability regulator. Impaired SYNGAP1 function leads to neurodevelopmental disorders (NDDs) characterized by intellectual disability (ID), epilepsy, and behavioral abnormalities. These variants disrupt Ras signaling, altering AMPA receptor transport and synaptic plasticity and contributing to cognitive and motor difficulties. Despite advancements, challenges remain in defining genotype–phenotype correlations and distinguishing SYNGAP1-related disorders from other NDDs, which could improve underdiagnosis and misdiagnosis. Brain Gene Registry: The Brain Gene Registry (BGR) was established as a collaborative initiative, consolidating genomic and phenotypic data across multiple research centers. This database allows for extensive analyses, facilitating improved diagnostic accuracy, earlier interventions, and targeted therapeutic strategies. The BGR enhances our understanding of rare genetic conditions and is critical for advancing research on SYNGAP1-related disorders. Conclusions: While no FDA-approved treatments exist for SYNGAP1-related disorders, several therapeutic approaches are being investigated. These include taurine supplementation, ketogenic diets, and molecular strategies such as antisense oligonucleotide therapy to restore *SYNGAP1* expression. Behavioral and rehabilitative interventions remain key for managing developmental and cognitive symptoms. Advancing research through initiatives like the BGR is crucial for refining genotype–phenotype associations and developing precision medicine approaches. A comprehensive understanding of SYNGAP1-related disorders will improve clinical outcomes and patient care, underscoring the need for continued interdisciplinary collaboration in neurodevelopmental genetics.

## 1. Introduction

### 1.1. Review of SYNGAP1

The human brain is the most complex organ in the body, regulating emotions, memories, and actions. At the core of its function are billions of neurons, which communicate via specialized structures called synapses. These synapses are dynamic and have the ability to adapt over time, supporting learning and memory [1]. Many biochemical pathways, including those involving essential proteins encoded by particular genes, are necessary for this malleability [2]. One gene, *SYNGAP1*, is critical in regulating synaptic function and plasticity. This review will explore the role of *SYNGAP1* in the brain and how its variants contribute to neurodevelopmental disorders (NDDs).

*SYNGAP1* is a gene encoding the cytosolic Synaptic Ras GTPase-Activating Protein (SYNGAP) [3]. SYNGAP1 was first identified in dendritic spines and interacts with key synaptic proteins, including PSD-95 and CaMKII, to regulate synaptic plasticity [4,5]. Both studies revealed that Syngap1 interacts with postsynaptic density protein 95 (PSD-95), a major scaffolding protein bound by several other PSD proteins in the synapse [6].

Syngap1 is a key regulator of neuronal excitability and stability. Research indicates that Syngap1 controls the activity of AMPA receptors, which are necessary for fast excitatory neurotransmission in the brain [1]. The loss of Syngap1 function disrupts the balance of excitation and inhibition, leading to hyperexcitability and further risk of epilepsy [7]. Variants in *SYNGAP1* result in NDDs with a phenotype that includes intellectual disability (ID), motor impairments, and epilepsy [3].

**Figure 1:**
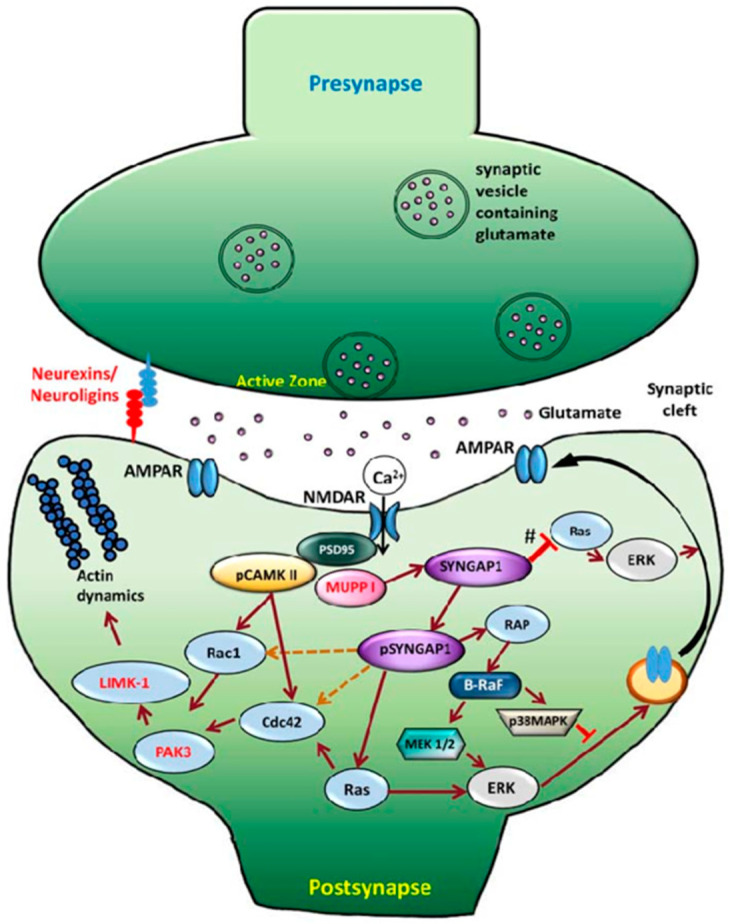
Schematic of synaptic signaling regulation pathway, linking CaMKII activity to phosphorylation of SYNGAP1 and its regulation of downstream effects [1]. *SYNGAP1* heterozygotes (symbolized as #) are unable to effectively inhibit Ras activation, resulting in increased AMPAR exocytosis to the post-synaptic membrane [1]. It is unclear how Syngap1 (pSYNGAP1) regulates Cdc42 and Rac1, as represented by the dotted orange lines [1]. Image from Jeyabalan & Clement (2016) [1].

Several studies have contributed to elucidating the regulatory pathway involving Syngap1. Glutamate activates NMDARs, triggering CaMKII phosphorylation, which activates Syngap1. This regulates Ras and Rap, leading to AMPAR endocytosis and reduced AMPAR presence at the postsynaptic membrane (Figure 1) [1].

Beyond individual studies, large-scale patient registries have become valuable for advancements in understanding rare genetic conditions. The Brain Gene Registry (BGR) was established to consolidate genomic and phenotypic information across multiple research centers, allowing for comprehensive genotype–phenotype correlation studies [8]. The BGR seeks to fill critical knowledge gaps in disorders like SYNGAP1-related ID by compiling patient data across various platforms. This collaboration improves diagnosis, patient care, and therapy development. In order to shape future research and treatment approaches, centralized databases such as the BGR will be essential as the profession shifts toward precision medicine [9].

### 1.2. Genotype and Phenotype

In individuals with a heterozygous variant in *SYNGAP1*, the ability of Syngap1 to inhibit Ras has been disrupted. Ras proteins act as binary molecular switches, cycling between guanosine triphosphate (GTP)-bound and inactive guanosine diphosphate (GDP)-bound states [10]. The dysregulation of Ras activity in SYNGAP1-deficient neurons not only elevates AMPAR exocytosis at the postsynaptic membrane, resulting in alterations in the dendritic spine, but also disrupts the MAPK/ERK signaling pathway, further impairing synaptic plasticity [3]. This alteration in dendritic spines disrupts neuronal growth and maturation, underlying cognitive and behavioral deficits. Therefore, research over the past decade has provided increasing evidence that *SYNGAP1* is a risk gene for NDDs, including ID. In 2009, the first de novo variants associated with ID in humans were identified [11]. Since then, over 200 patients with loss-of-function variants (e.g., missense, frameshift, etc.) in *SYNGAP1* have been identified using genetic sequencing [6].

Variants associated with ID have been identified along the entire length of *SYNGAP1*, with most variants occurring from exons 3–17 [6]. SYNGAP1-related disorders follow an autosomal dominant inheritance pattern. Diagnosis is based on heterozygous pathogenic variants or 6p21.3 deletions in individuals with ID [12]. Homozygous variants are fatal. While most *SYNGAP1* variants are de novo, affected offspring in familial cases with vertical transmission from mosaic parents have been described [12]. Notably, *SYNGAP1* loss-of-function variants have a reported incidence of 1–4/10,000 individuals, or 0.5–1.0% of all ID cases [13]. However, a significant gap exists between the predicted incidence and the number of patients documented in the literature.

Underdiagnosis stems from clinician awareness, phenotypic variability, and symptom overlap with other NDDs. SYNGAP1-related disorders share features with other NDDs, such as Rett syndrome and SCN2A disorders, complicating diagnosis. Improved molecular and phenotypic characterization will be critical for this distinction [9].

Previous studies have found that patients with variants in exons 1 to 5 have milder ID than those with variants in exons 8 to 15 [14]. Moreover, another study found that variants in exons 4 and 5 increase patients’ risk of developing refractory epilepsy [15]. Further research remains necessary, specifically to define the phenotypic range and comprehend the effects of different *SYNGAP1* mutations on function and clinical presentation. A deeper comprehension of these connections will increase accurate diagnoses, allow for earlier interventions, and improve genetic counseling for impacted families.

No genotype–phenotype correlations have been published; however, there may be loci within *SYNGAP1* that are prone to mutation [16]. For example, a missense variant (p.Pro562Leu) in the RASGAP domain has been reported four times in ClinVar, in addition to a nonsense variant (p.Arg143*) [15,16]. Both have been reported in patients with severe ID and delay [17].

Patients with *SYNGAP1* variants exhibit cognitive impairments, with learning, memory, and executive function deficits. These conditions stem from the dysregulation of synaptic plasticity due to defective Ras signaling [6]. The specific variant location within *SYNGAP1* may influence the extent of cognitive impairment, as previously described. Epilepsy is another predominant feature, with varying phenotypes such as generalized, focal, and myoclonic seizures commonly reported [17]. Studies have provided critical insights into the epileptogenic nature of SYNGAP1-related disorders. For instance, hyperexcitability and lowered seizure thresholds can be caused by haploinsufficiency, which prevents the production of a normal gene product [12]. The comprehensive phenotype study by Wiltrout, Brimble, and Poduri (2024) also confirmed the high prevalence of epilepsy among affected individuals [18].

Recent research has highlighted the significant impact of *SYNGAP1* mutations on sensory processing. Patients have experienced hypersensitivity to auditory processing, visual, tactile, gustation, and proprioception stimuli [19]. These sensory deficits may contribute to behavioral challenges, including emotional dysregulation, anxiety, and difficulty handling change [16]. Additionally, a study led by Dr. Gavin Rumbaugh of Scripps Institute identified abnormalities in sensory information processing, suggesting that dysfunction in cortical circuits underlies these issues [20].

Furthermore, approximately 50% of individuals with SYNGAP1 syndrome meet the criteria for ASD [12]. Social communication deficits, repetitive behaviors, and restrictive interests are frequently reported [16]. Furthermore, behavioral issues are prevalent [19]. Improved molecular and phenotypic characterization is required to improve diagnostic standards and therapeutic strategies as SYNGAP1-related diseases and ASD have similarities [9]. Additionally, motor deficits are frequently observed, including hypotonia, gait abnormalities, and delayed gross and fine motor development [3]. Some individuals exhibit dysmorphic features, although these are generally mild and nonspecific.

Published cases have also provided valuable insight into the variable presentation of SYNGAP1 syndrome. Of note, hyperactive catatonia has been identified in two patients with *SYNGAP1* variants and ASD [21]. Behavioral issues, including self-injurious behaviors and aggression, have been described in the adolescent and adult phenotypes [22] and in patients with deleterious variants [16]. However, catatonia as a cause of these behavioral disturbances and as a manifestation of SYNGAP1 syndrome remains under-explored.

Additionally, a mitochondrial phenotype of SYNGAP1 syndrome is being considered. There are overlapping features between SYNGAP1 syndrome and mitochondrial disorders, such as intractable epilepsy, ASD, excessive fatigue, and altered biochemical markers. To our knowledge, no published case has linked the two. A patient with a pathogenic variant in *SYNGAP1* and a non-pathologic mitochondrial variant had a normal newborn screen. However, delayed achievement of developmental milestones, sensory and social issues, and seizures with onset after weaning were apparent. EEG suggestive of encephalopathy with generalized epilepsy was reported, and the patient was also found to have elevated levels of lactate and alanine and decreased levels of citrulline [23]. It is possible that mitochondrial dysfunction may be part of the continuously evolving phenotypic spectrum of SYNGAP1 syndrome. The patient improved with MCT oil and taurine, showing a gain in speech and motor function, as well as a decrease in the number of daily seizures [23].

### 1.3. Treatment

There are no FDA-approved treatments for SYNGAP1-related disorders, but various symptomatic and exploratory therapeutic approaches are being investigated. As of now, the two most common treatment options are taurine and a ketogenic diet. Taurine, a naturally occurring amino acid, has been explored due to its role in modulating neuronal excitability [6]. Studies suggest that taurine supplementation may help stabilize neuronal hyperexcitability by enhancing inhibitory neurotransmission and modulating calcium homeostasis. The relationship between changes in the local concentration of amino acids in different brain structures and seizures is not well recognized, and further clinical trials are needed to determine taurine efficacy and safety in individuals with *SYNGAP1* mutations.

The ketogenic diet, which is high in fat and low in carbohydrates, has been broadly used to treat refractory epilepsy [18]. This diet promotes ketone body production, which has neuroprotective and anticonvulsant effects. A medium-chain triglyceride diet was also introduced as an alternative to the ketogenic diet, and due to their smaller length, medium-chain fatty acids (MCFAs) can be metabolized by astrocytes in the brain, bypassing eventual transport to the mitochondria for β-oxidation [24]. By having a faster metabolism than long-chain fatty acids, MCFAs may be superior in supplementing the energy needs in epileptogenic brain areas [24].

Given haploinsufficiency, another exploratory therapeutic approach has focused on upregulating Syngap1 to levels seen in healthy individuals. Dawicki-McKenna et al., 2023, show that polypyrimidine tract binding protein-2 (PTBP-2) targets and binds to *SYNGAP1* mRNA, promoting alternative splicing and nonsense-mediated decay [25]. They also found that antisense oligonucleotides disrupt PTBP-2 binding, redirecting splicing and increasing *SYNGAP1* mRNA and protein expression [25].

Due to SYNGAP1 syndrome presenting with a spectrum of symptoms, there is a wide variety of treatments available to manage them. For epilepsy, antiepileptic drugs can be effective in some individuals, though drug-resistant epilepsy remains a challenge [17]. In fact, Perampanel is being explored as a therapeutic option and, potentially, as an adjunctive treatment with other anti-seizure medications [26]. Nortriptyline, a tricyclic antidepressant, is also being explored for its anticonvulsant effects [27]. Regarding developmental delays, speech therapy, occupational therapy, and behavioral therapies such as ABA are widely used to improve cognitive and motor skills [3]. Furthermore, for behavioral issues, psychiatric support and behavioral therapy are often necessary to manage hyperactivity, aggression, and emotional dysregulation [19]. Improved recognition and diagnosis of SYNGAP1-related disorders will facilitate earlier interventions and targeted therapeutic strategies, ultimately improving patient outcomes [8].

## 2. Brain Gene Registry

### 2.1. Background

Our understanding of SYNGAP1 syndrome and other NDDs evolves with each new patient case. However, individualized case reports can limit broader insights into genetic conditions. The BGR was established to address this by collecting data across multiple centers, allowing for a large-scale analysis.

Formed in 2022 as a collective effort among researchers affiliated with Intellectual and Developmental Disabilities Research Centers (IDDRCs) across the United States, the BGR was funded by the National Institutes of Health’s National Center for Advancing Translational Sciences. The registry serves as a centralized repository with paired genomic and phenotypic data to advance and increase understanding of the impact of rare gene variants in intellectual and developmental disabilities. The BGR supports gene curation, refines genotype–phenotype correlations, and facilitates the development of targeted therapies [9]. This multi-institutional collaboration spans at least 13 sites, enhancing the ability to conclude rare variants by moving beyond case studies with small sample sizes. Instead of relying on an “N of 1” approach, where only one individual acts as their control by comparing different treatments administered to them over time, the BGR aggregates patient data to enable the generation of statistically significant associations.

The cumulative burden of rare diseases is substantial, with over 7000 identified rare diseases affecting an estimated 25–30 million Americans [28]. An estimated 80% of rare diseases have a genetic etiology, and advancements in genomic sequencing have enabled the possibility and progression of new therapies or treatments [28]. However, the efficiency of these advancements is set back by single-disease approaches, which fail to identify shared mechanisms, lack coordination of outcome measures across overlapping disorders, and have limited data sharing across institutions, which restricts broad comparative studies [28]. The BGR fosters collaboration among investigators, patient advocacy groups, and gene curation panels, helping identify commonalities and distinctions in phenotype and etiology.

By consolidating data, the BGR aims to address critical gaps in care, such as delays in diagnosis, misdiagnosis, limited access to knowledgeable clinicians, and the lack of coordinated care. Additionally, the BGR promotes the development of more effective therapies and management strategies. The utility of electronic medical record (EMR) data for rare disease research has not been well studied due to the challenges of accumulating data across institutions [8]. The BGR maximizes recruitment by utilizing its network of IDDRCs and identifying individuals who might have been overlooked otherwise. While the BGR’s remote consent process facilitates broader patient recruitment, it has certain challenges. It is susceptible to survey response biases, where subjective interpretations and self-report inaccuracies can affect data reliability. Additionally, missing data from non-respondents may limit the completeness of genotype–phenotype correlations. Technical barriers, such as poor internet access, could further impact participation/data quality. Addressing these challenges will be crucial to maximizing the BGR’s effectiveness.

Notably, obtaining site-specific statistics from platforms like CIELO could provide key insights into participation trends and data completeness. For example, tracking the number of participants who have fully completed study requirements versus those still in progress may help clarify data collection strategies. Additionally, CIELO’s statistics, such as the percentage of RNAPs completed, EHRs uploaded, and GenomeConnects submissions, could help assess and reduce data gaps within the BGR’s remote data collection. For instance, there are currently 674 patients enrolled in the BGR, of which 100% have genetic reports with a primary gene and 95% have RNAP feedback reports available through CIELO. Sixty-four percent have EHR available. Therefore, there may be a delay in data entry or sharing; however, these metrics are useful for determining data completeness and enable study sites to proactively follow data updates.

Moreover, clinicians may diagnose epilepsy without further investigation into its genetic origins. This results in the underdiagnosis of SYNGAP1 syndrome and other genetic disorders. This is also attributed to the lack of a current, comprehensive, standardized, and validated resource of phenotypic information on *SYNGAP1*. All of these reasons underscore the need and utility of the BGR.

### 2.2. Expanding Current Databases

ClinVar, a commonly used database that makes variants and their classifications (i.e., pathogenic/likely pathogenic, benign/likely benign, and variant of unknown significance) publicly available, allows geneticists and other specialists to access variant information. However, there are limitations. For example, if multiple genetic laboratories submit conflicting classifications, ClinVar categorizes the variant as having “conflicting interpretations of pathogenicity” [9]. Additionally, there is no systematic method for clinicians or consented individuals to be recontacted by those interested in a specific variant [9]. Patients with variants of unknown significance (VUS), in particular, may be lost to follow-up if they cannot be contacted about re-classification of their variant to pathogenic/likely pathogenic, especially if their referring physician is no longer involved in their care.

Furthermore, although genetic laboratories are encouraged to contribute variant data to ClinVar, participation can be inconsistent due to lab-dependent workflows and protocols [9]. When data are submitted, they often lack or restrict the amount of phenotypic information, which can be subject to error or incompletion [9]. Therefore, the BGR was formed in response to these pitfalls by standardizing phenotypic data collection and providing a structured mechanism for data sharing and updates.

To date, Chopra et al. (2024) found that 42.5% of variants collected by the BGR were not recorded in ClinVar and 34.6% were absent from other rare-disease databases [9]. Discrepancies were observed in nine cases where the BGR classification, as reported by participants, differed from that recorded in ClinVar [9]. Notably, three of these discrepancies involved major classification differences, like a VUS reported to the BGR but classified as likely benign (LB) or pathogenic (P) in ClinVar [9]. In these cases, the BGR report predated ClinVar’s, indicating that participants may have been unaware of updated classifications [9]. In another three cases, the BGR report date was more recent than ClinVar’s, highlighting the registry’s ability to provide updated variant classification information [9].

Additionally, Cordova et al. (2024) reported that BGR information expanded the understanding of the phenotypic spectrum associated with ASH1L-related disorder [29]. Baldridge et al. (2024) further noted that, as of April 2024, data from the BGR had been used in the curation of 36 gene–disease relationships by the Intellectual Disability/Autism Gene Curation Expert Panel (ClinGen GCEP) [8]. These findings show how the BGR can contribute to novel genomic data. In general, BGR investigators are continuing to analyze registry data and additional publications are underway.

**Figure 2:**
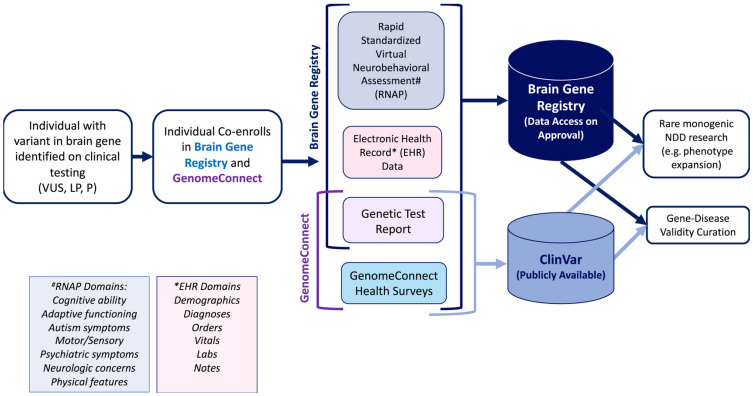
Structure and workflow of the Brain Gene Registry (BGR) and comparison of data completeness between the BGR and ClinVar, showing the proportion of unique variants captured by the BGR versus ClinVar [9]. The information collected by the RNAP is listed under “#RNAP Domains”, and the information included in the EHR is listed under “*EHR Domains”. EHR, electronic health record; LP/P, likely pathogenic/pathogenic; NDD, neurodevelopmental disorder; RNAP, rapid neurobehavioral assessment protocol; VUS, variant of uncertain significance [9]. Image from Chopra et al. (2024) [9].

Furthermore, to enhance accessibility and accuracy, BGR participants are co-enrolled in Genome Collect, an NIH-related registry that submits genomic and health survey data to ClinVar. This allows for public accessibility, participant connection, and recruitment opportunities for additional studies. Over time, data from these registries will eventually merge, as shown in Figure 2 [9].

### 2.3. How the BGR Will Inform Clinical Trial Design

The BGR facilitates clinical trial development for emerging therapies by aggregating standardized genotype–phenotype data. The registry provides a crucial resource for designing targeted interventions, identifying patient cohorts, and tracking longitudinal outcomes.

The Rapid Neurobehavioral Assessment (RNAP) is a remote assessment tool used by the BGR and it includes validated questionnaires and behavioral surveys. It systematically evaluates key function domains relevant to the comprehensive phenotypic characterization of NDDs and sub-clinical neurodevelopmental disorder traits [9]. Domains such as cognition, adaptive functioning, motor/sensory, autism symptoms, psychiatric symptoms, physical characteristics, and neurological concerns are evaluated. The RNAP typically takes 60 min to complete, and the administering person must complete training and demonstrate proficiency through the BGR. The questionnaires are selected based on age and scored electronically. A significant benefit of having access to the patient’s EMR includes comparing the questionnaire scores with the patient’s history and previously completed evaluations. This almost serves as an internal quality assurance, further strengthening gene curation.

Our site at the Children’s National Hospital chose to incorporate an IRB-approved (PRO15723) survey assessing patient experience with the RNAP. Notably, responses were not confined to a specific gene, such as *SYNGAP1*; instead, surveys were sent to all patients who had completed the study requirements at our site. The questions focused on difficulties with the remote format, preference for remote versus in-person, and general feedback. While the study is still ongoing, we have received responses from a total of nineteen families. Most of them (94.7%) did not report difficulties with the remote setup, and the one exception struggled to complete the assessment virtually. All nineteen families reported favoring the remote aspect, with fourteen rating their experience as excellent, four as good, and one as fair. Some families provided general impressions of their experience, including the “assessment was thorough and easy to complete”. One parent reported that they preferred both in-person and online formatting; however, they admitted that an online version is easier for them.

We anticipate current and future feedback will help inform the use of remote assessment in clinical trials. One outcome of the COVID-19 era was the wider implementation of telemedicine; however, the utility of remote medicine in the rare-disease space has been increasingly relevant, even before the onset of COVID-19 social distancing policies. Clinical trials for rare genetic diseases have been a source of hope for families. They may also be a source of burden. The associated financial and psychological burdens challenge recruitment, enrollment, and study retention. For example, clinical trial sponsors will designate how patient families can use their accommodations, and often, in rare diseases, this does not include travel between home and the trial site. As a result, families are required to move near the trial site for the study’s duration and likely in open-label extension. The patient’s parents and/or caregivers must also navigate employment changes and, potentially, new school transfers for the patient and their siblings. The sacrifices and life adjustments involved in trial participation could be minimized with the implementation of remote assessments. By incorporating them into its study design, the BGR is helping to further greater adoption of a “minimally disruptive and compassionate clinical research” approach [30].

### 2.4. Site Enrollment, Data Security & Patient Privacy

As for enrollment in the BGR, subjects are recruited across medical specialties at all sites. In order to be eligible, the variant must be reported by a clinical laboratory with a Clinical Laboratory Improvement Amendment (CLIA) certification and be classified as VUS, likely pathogenic, or pathogenic according to professional guidelines [9]. Eligibility is determined by variant only, regardless of the testing indication and subject phenotype [9]. Participants will be able to share their genetic testing results with the BGR study team using a HIPAA-secure, deidentified folder [31]. If they are found to have an eligible variant and wish to be enrolled, informed consent is obtained and electronic medical record (EMR)-derived phenotypic data are shared with the BGR, including any clinical testing, dietary information, and treatments. To ensure the privacy of participants, any research will be covered by a Certificate of Confidentiality from the federal government [31]. If they choose to not enroll, their data will be deleted [31]. Once enrolled, the subject will be paired with a site (i.e., Children’s National Hospital) to participate in the RNAP. The subject’s deidentified EMR data will also be shared with the site using a secure database. Of note, there are limited publicly available data on the BGR registry website (please see Data Availability Statement).

## 3. Conclusions

*SYNGAP1* is essential for learning, memory, synaptic plasticity, and neuronal excitability [6,7]. A range of NDDs, including ID, epilepsy, problems in sensory processing, and behavioral difficulties, are caused by variations in this gene [12,15]. Despite the increasing recognition of SYNGAP1-related disorders, significant gaps remain in understanding the full genotype–phenotype correlations, contributing to underdiagnosis and variability in clinical presentations [14,18].

Efforts like the BGR are crucial in addressing these gaps by accumulating patient data across multiple institutions, improving genetic diagnoses, and fostering targeted therapeutic development [8,9]. Although symptomatic treatments such as behavioral therapy, dietary changes, and antiepileptic medications aid in symptom management, recent studies on upregulating *SYNGAP1* offer encouraging treatment options for the future [25,26]. Further studies are needed to refine diagnostic criteria so that underdiagnosis does not occur as frequently, explore personalized interventions, and advance therapeutic options. This will ultimately improve outcomes for individuals affected by SYNGAP1-related disorders.

## Data Availability

There are limited publicly available data on the registry website. Identifiable data that the Children’s National Hospital collects are unable to be requested to protect the confidentiality of the participants.

## Abbreviations

The following abbreviations are used in this manuscript:

NDD	Neurodevelopmental Disorder
ID	Intellectual Disability
BGR	Brain Gene Registry
